# Intraspecific Autochthonous and Allochthonous Resource Use by Zooplankton in a Humic Lake during the Transitions between Winter, Summer and Fall

**DOI:** 10.1371/journal.pone.0120575

**Published:** 2015-03-12

**Authors:** Martin Berggren, Ann-Kristin Bergström, Jan Karlsson

**Affiliations:** 1 Department of Physical Geography and Ecosystem Science, Lund University, Lund, Sweden; 2 Department of Ecology and Environmental Science, Umeå University, Umeå, Sweden; University of Yamanashi, JAPAN

## Abstract

Seasonal patterns in assimilation of externally produced, allochthonous, organic matter into aquatic food webs are poorly understood, especially in brown-water lakes. We studied the allochthony (share biomass of terrestrial origin) in cladoceran, calanoid and cyclopoid micro-crustacean zooplankton from late winter to fall during two years in a small humic lake (Sweden). The use of allochthonous resources was important for sustaining a small population of calanoids in the water column during late winter. However, in summer the calanoids shifted to 100% herbivory, increasing their biomass several-fold by making efficient use of the pelagic primary production. In contrast, the cyclopoids and cladocerans remained at high levels of allochthony throughout the seasons, both groups showing the mean allochthony of 0.56 (range in mean 0.17-0.79 and 0.34-0.75, for the respective group, depending on model parameters). Our study shows that terrestrial organic matter can be an important resource for cyclopoids and cladocerans on an annual basis, forming a significant link between terrestrial organic matter and the higher trophic levels of the food web, but it can also be important for sustaining otherwise herbivorous calanoids during periods of low primary production in late winter.

## Introduction

Lake ecosystems receive external (allochthonous) input of organic matter from the surrounding terrestrial landscape, at loading rates that may widely exceed the internal (autochthonous) photosynthetic production in the water column [1–3]. The extent to which such allochthonous organic matter can be assimilated into aquatic food webs is debated [4, 5], mainly because the mechanisms behind allochthonous resource use in consumers have for long been unclear. Most of the allochthonous material arrives to lakes as dissolved organic carbon (DOC) that can mainly be used by heterotrophic bacterioplankton [6]. In turn, many feeding experiments suggest that bacterioplankton represent a poor single food source for zooplankton [7, 8], as does other potentially ingestible particles of terrestrial origin, e.g., fragments of leaves, litter or soil [5, 9], due to their lack of biochemicals (i.e. polyunsaturated fatty acids and sterols) which are critical for somatic growth and reproduction [10].

Nevertheless, isotope mixing models have systematically provided strong support for a general tendency of significant allochthony (share biomass of terrestrial origin) among many abundant zooplankton taxa, especially crustaceans [11–13]. Part of the explanation to this paradox could be found in recent laboratory work, showing that zooplankton which feed on mixed diets can use the terrestrially-derived resources to extract carbon, energy and nutrients (particularly phosphorus from bacterioplankton [14, 15]), provided that sufficient amounts of essential fatty acids and sterols are obtained in parallel from phytoplankton [9, 16]. Further, if terrestrial particles and bacterioplankton first are consumed by heterotrophic microzooplankton grazers (ciliates or flagellates), which in turn are consumed by metazoan zooplankton, then theoretically this represents an upgrade of the food quality in which nutritionally important fatty acids are added [17]. A similar upgrade in food quality should happen when allochthonous organic matter is first assimilated by phagotrophic [18] or osmotrophic [19] mixotrophic phytoplankton.

In this regard, there is already today knowledge about both the existence of zooplankton allochthony and its potential mechanistic underpinnings. However, there is a lack of field studies that link the different mechanisms to the *in situ* regulation of allochthony in different taxa and across resource gradients both in space and time. The relevance of such studies was recently highlighted in a survey of 18 lakes in Canada, showing that the allochthony regulation clearly was expressed differently among major crustacean orders [4]. For example, allochthony in cyclopoids was positively correlated to high concentrations of bioavailable DOC and to bacterioplankton production (BP), indicating that cyclopoids generally were utilizing DOC-based food chains. In contrast, allochthony in calanoids was related to changes in the bulk particle composition, with the highest values in lakes with predominantly terrestrially-derived particulate organic matter pools [4].

Hypothetically, similar differences in allochthony regulation should be expressed also on temporal scales. However, the seasonality of zooplankton allochthony has been addressed only in a few studies, almost exclusively targeting oligotrophic clear-water lakes [20–22]; DOC-rich brown-water systems, where allochthony is believed to be most widespread [23], remain to a large extent unexplored. Moreover, virtually no hypothesis-driven study has evaluated intraspecific seasonal allochthony patterns in relation to potential mechanisms (e.g., particle ingestion, bacterivory, mixotrophy, microzooplankton food chains). Expanding on previous findings [4], we here bring forward the idea that selective use of autochthonous and allochthonous resources, respectively, is an important part of the resource niche separation between calanoid and cyclopoid copepods in brown unproductive lakes. We hypothesize that calanoids act as herbivores when phytoplankton is available [4], while the cyclopoids continue to use allochthonous resources also during periods of significant primary production. The rationale for this hypothesis is that cyclopoids can avoid competition with calanoids for phytoplankton by using their raptorial style to feed on bacterivorous microzooplankton and other heterotrophs [24, 25], which connects cyclopoids to heterotrophic food chains fueled by allochthonous DOC [26].

We tested this idea in a two-year seasonal study of an unproductive boreal lake in northern Sweden, where previous studies [2, 27] have found BP to be several-fold higher than the primary production (PP). It was predicted that allochthony in calanoids would be negatively correlated to the relative abundance of phytoplankton particles, but that allochthony in cyclopoids rather should be regulated by positive correlations to DOC concentrations and BP rates. Cladocerans, being relatively non-specific filter feeders, were expected to show a high degree of allochthony throughout the seasons in this type of lake. Our results largely confirm these hypotheses and expectations, and further show that variability in zooplankton allochthony, temporal and intraspecific, within a single lake can be as large as the variability in community-level zooplankton allochthony found in previous surveys of lakes across broad landscape gradients.

## Materials and Methods

### Study Site

We selected the small (0.05 km^2^) unproductive brown-water lake Övre Björntjärn (a.k.a. Upper Bear Lake; 64°7′23.53″N, 18°46′43.04″E, northern Sweden) for this study. This lake was the single most net heterotrophic system of the unproductive lakes compiled across a wide DOC gradient by Jansson et al. [27]. In another study is was estimated that allochthonous organic matter contributed to 99.9% of the organic matter loading to the lake, ≥96% of the ecosystem respiration, ≥92% of BP, and roughly half of the biomasses of crustacean zooplankton and of fish [2].

Övre Björntjärn is characterized by a dynamic hydrology where inputs of fresh DOC come in pulses during high flow episodes, in turn regulating the variability in BP [28]. The lake has, as other humic lakes in this region (see Drakare et al. [29]), a short stratified summer season and typically a single phytoplankton peak in summer (no spring blooms have been noted). Further, because of the brown and relatively deep nature of the lake (max 8 m, high littoral slope), the autochthonous production is mainly performed by phytoplankton, while benthic primary production is one order of magnitude lower in comparison [2]. The lake catchment consists of coniferous forest and *Sphagnum* peat wetlands.

### Sampling and Analyses

Sampling for zooplankton stable H isotopes was performed on 10 dates in 2009 and 5 dates in 2011, by vertically hauling a plankton net through the top 4–5 m of the deepest point of the lake. No specific permissions were required for sampling of these invertebrate animals at the study site (64°7′23.53″N, 18°46′43.04″E). The field studies did not involve endangered or protected species. The zooplankton were stored in filtered lake water for gut evacuation 12–24 h, and then separated by hand into three fractions: calanoid copepods (*Eudiaptomus* sp.), cyclopoid copepods (*Cyclops* spp.) and cladocerans (mainly *Ceriodaphnia* sp., sometimes also *Daphnia*, *Bosmina* and *Holopedium*). Biomass of zooplankton was determined using length to dry-weight regressions applied to microscopically assessed length measurements of each taxon [30]. Water column integrated values (mg m^-2^) were calculated by dividing the sampled biomass by the surface area of the plankton net. Alternatively, on two sampling occasions in late winter 2009, biomasses were obtained by sorting, drying and weighing zooplankton which represented a known volume of lake water. The carbon content of dry weight was assumed to be 48% [31].

Phytoplankton net primary production (PP) was measured at 5–6 depths of the photic layer (0–2 m; plus deeper controls) for 4 h around noon according to the standard ^14^C incorporation method [32]. Measured primary production was converted to daily whole-lake values (mg m^-2^ d^-1^) as described elsewhere [33]. Bacterioplankton production (BP) was measured both in epilimnetic and hypolimnetic samples using a slightly modified version [33] of the [3H]-leucine incorporation method [34]. For each date, the epilimmnetic and hypolimnetic BP rates were multiplied with the respective epilimmnetic and hypolimnetic whole-lake water volumes, and divided by lake area to generate daily whole-lake BP values (mg m^-2^ d^-1^). The upper and lower halves of the metalimnetic layer (temperature changes >1°C m^-1^) were considered to belong to the epilimnion and hypolimnion, respectively.

At epilimnetic (ca 1 m) and hypolimnetic (ca 5 m) depths, respectively, at the deepest part of the lake, the bacterial abundance, Chl-a, δ^2^H of water, DOC and particulate organic carbon (POC; selected dates) were sampled. DOC (0.45 μm-filtered) was analyzed on a TOC-VcPH (Shimadzu, Japan). POC was measured by filtering known water volumes through pre-combusted (550°C, 4h) 25mm GF/F glass fiber filters (Whatman, Ann Arbor, MI, USA). Filters were stored frozen until analyses, and then analyzed using a Costech ECS 4010 elemental analyzer (Costech International, Italy). Water for δ^2^H analysis was filtered (0.2 mm) and stored in airtight glass bottles without air bubbles until analysis. Chl-a was collected on Whatman GF/F filters, extracted for 24 h with 95% ethanol, and measured with a luminscence spectrometer (Perkin Elmer LS45). Phytoplankton biomass was calculated assuming a Chl-a to carbon conversion factor of 50 [35]. Samples for bacterial counts were fixed with filtered (0.2 μm) glutaraldehyde, and then filtered onto black 0.2 μm polycarbonate filters, stained with acridine orange and photographed (nine pictures per sample) under epiflourescence microscopy. Bacterial biomass was assessed using the image analysis system LabMicrobe [36], assuming a biovolume to carbon biomass factor of 0.308 pg C μm^-3^ [37].

Water column integrated values of DOC, POC, phytoplankton biomass and bacterial biomass (mg C m^-2^) were calculated using the same procedure as described above for BP. However, since there was no significant difference in the bacterial biomass per liter between the epilimnion and the hypolimnion of 2009 (paired t-test, *t* = -0.4, *p* > 0.33, *n* = 9), only epilimnetic bacterial samples were counted in 2011 and assumed to represent the whole water column. In addition, since the amount Chl-a in the mixed layer was eight times higher than that in the hypolimnion (average of 9 dates when hypolimnetic Chl-a could be detected), we considered the hypolimnetic Chl-a to be negligible in both years.

Zooplankton samples for isotopic analysis were freeze-dried or dried at 65°C, homogenized and transferred to silver capsules. Analyses of the δ^2^H of nonexchangeable H were carried out at the Colorado Plateau Stable Isotope Laboratory, Northern Arizona University, following Doucett et al. [38]. Organic matter samples and standards were equilibrated with local water vapor to correct for exchangeable H. Solid samples were analyzed by means of pyrolysis, and the isotopic composition of H_2_ gas was measured using isotope ratio mass spectrometry. The δ^2^H of water samples was analyzed by headspace equilibration with H_2_ gas and a platinum catalyst using isotope ratio mass spectrometry. The δ^2^H data are expressed in per mil (%) notation relative to Vienna Standard Mean Ocean Water.

We estimated the water discharge from the study catchment using daily means of specific discharge from the nearby Krycklan catchment (50 km northeast), where stream water levels were recorded continuously [39]. Registered water levels at the inlet of Övre Björntjärnen on a total of 18 dates, 2007–2009, demonstrated a strong correlation with the water level in the Krycklan stream (*r* ^2^ = 0.85, *n* = 18, *p* < 0.01), indicating that the discharges at the two sites are proportional to each other. Additionally, the magnitude of the specific discharge is similar between the two sites (for further explanation and references, see [2]).

### Allochthony Assessment

Allochthony assessments in recent literature have increasingly been based on stable isotope mixing models that simultaneously handle several isotope ratios (e.g., δ^2^H, δ^13^C and δ^15^N) and several sources (e.g., benthic algal, phytoplanktonic and allochthonous) [11, 40]. However, for boreal low-productive lakes, Berggren et al. [4] found that there is a very strong agreement (r^2^ = 0.95) between the allocthony derived from an algebraic 2-source δ^2^H mixing model, and allocthony derived from a more advanced multi-isotope model which differentiates between phytoplanktonic and benthic algal production. Therefore, we estimated allochthony using a simple autochthonous-allochthonous δ^2^H mixing model [2, 41] (equation 1), based on the δ^2^H of zooplankton samples (δ^2^H_zoo_), the allochthonous source (δ^2^H_allo_), the autochthonous source (δ^2^H_auto_), and the ‘δ^2^H enrichment’ in zooplankton due to incorporation of H from ambient lake water [42].

$$allochthony\;=\;\frac{\delta^{2}H_{zoo}-\delta^{2}H\;enrichment-\delta^{2}H_{auto}}{\delta^{2}H_{allo}-\delta^{2}H_{auto}}$$ (1)

For each trophic level, we assumed that dietary water contributed to a fraction of consumer H (ω) of 0.15, following Berggren et al. [4]. Total contribution of water-derived H (ω_tot_) was calculated according to equation 2, where τ is the number of trophic levels between the source- and consumer-level. We assumed τ = 1 for all cladoceran samples [43] and estimated τ for calanoids and cyclopoids to 1.5 and 2, respectively, from their mean δ^15^N values in the lake in relation to the mean δ^15^N of cladocerans (J Karlsson and A-K Bergström unpubl. δ^15^N data), assuming a per-trophic-level stable nitrogen isotope fractionation (ΔN) of 3.4‰ [44]. The δ^2^H enrichment for each sample could then be calculated (equation 3).

$$\omega_{tot}\;=\;1-{\left(1-\omega \right)}^{\tau }$$ (2)

$$\delta^{2}H\;enrichment\;=\;\frac{\delta^{2}H_{zoo}-({\delta^{2}H_{zoo}-\omega }_{tot}∙\delta^{2}H_{water})}{1-\omega_{tot}}$$ (3)

We assumed an δ^2^H_allo_ of -134.5‰ ± 12.3‰ (mean ± *SD*) from the measurements on terrestrial sources in the form of peat, spruce and pine soils of the catchment (*n* = 8) made by Karlsson et al. [2]. The δ^2^H_auto_ was calculated as δ^2^H of H_2_O minus the stable hydrogen photosynthetic fractionation factor, i.e., difference in δ^2^H between phytoplankton and H_2_O. Again, we used data from Karlsson et al. [2], showing that δ^2^H (and thus the photosynthetic fractionation factor) was not significantly different between periphyton (benthic algae) and phytoplankton from the lake, regrown in the laboratory. Therefore, we used the mean fractionation of all four phytoplankton regrowth experiments and three periphyton samples (-144.5‰ ± 15.7‰) from Övre Björntjärnen available from 2009 (see details in Karlsson et al. [2]).

Further, we estimated the allochthony of the total crustacean community by calculating the biomass-weighted mean allochthony of the different groups. To address uncertainties, we calculated allochthony in alternative scenarios by manipulating the allochthonous and autochthonous end members for δ^2^H by ± 1 *SD* and by varying ΔN (2.42–4.42 [44]) and ω (0.10–0.22 [45]).

## Results

**Fig 1:**
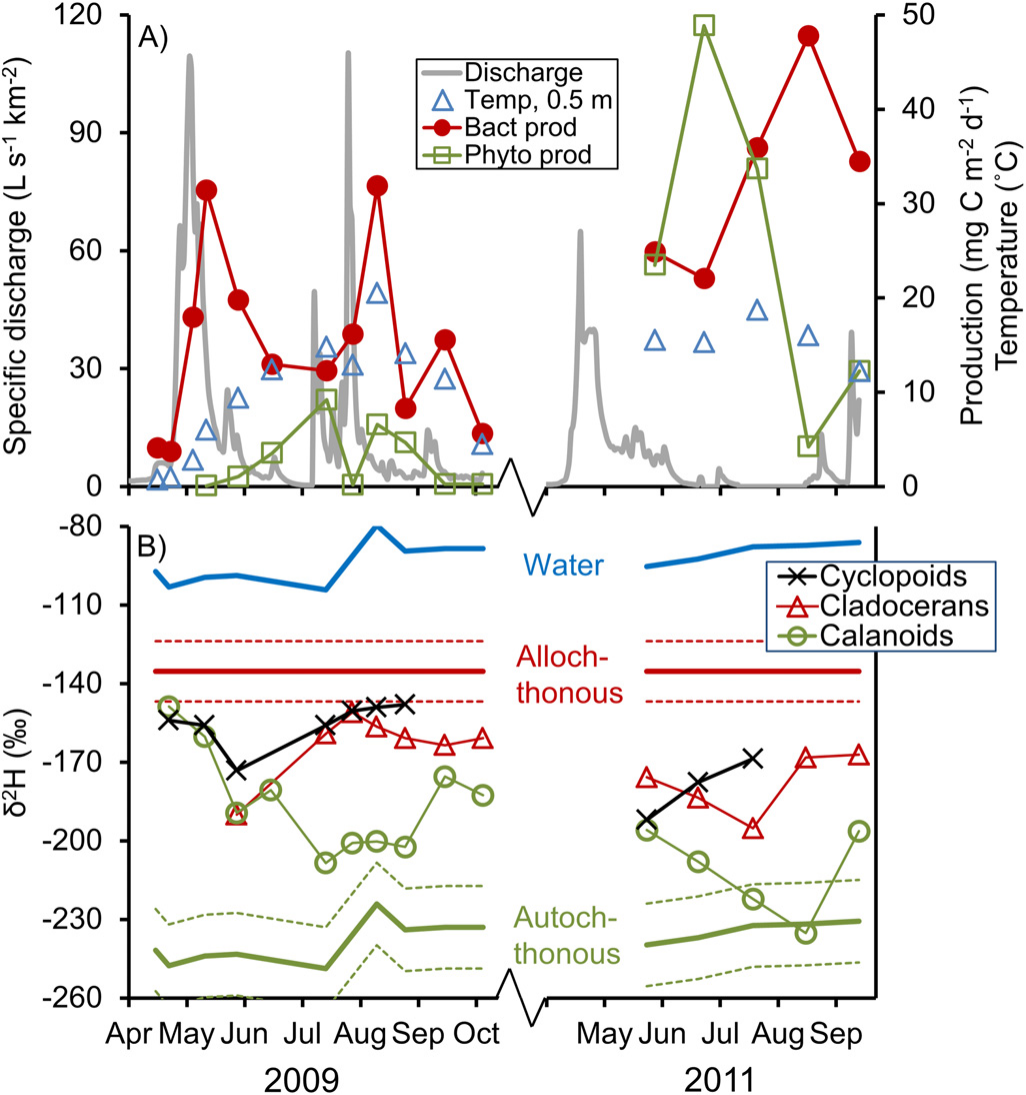
Seasonal patterns in lake Övre Björntjärn from spring to fall. (A) Phytoplankton net primary production (PP) and bacterioplankton production (BP) shown together with catchment water discharge and lake water temperature; (B) stable hydrogen isotope ratios (δ^2^H) of water, zooplankton groups and allochthonous and autochthonous organic matter. Dashed lines show ± 1 *SD* of allochthonous and autochthonous organic matter.

The annual winter ice disappeared around the snowmelt discharge peak in early May (2009) or mid-April (2011), and subsequently the mixed layer temperature of the lake started to rise from approximately 0°C under ice to maximum observations in the range of 15–20°C in June, July and August (Fig. 1a). In 2009, the production processes showed patterns typical for this site (see study site description), with mean BP (15 mg C m^-2^) being several times higher than mean PP (3 mg C m^-2^), and absolute BP values increasing strongly after high flow events in spring and late summer (Fig. 1a). However, in 2011 discharge was low throughout the season and the lake was unusually productive, with BP and PP showing similar mean magnitudes (both *ca* 30 mg C m^-2^). The PP peaked in July during both years, but was clearly negatively affected by a rain storm event in the late July of 2009, which caused temporary drops in PP (Fig. 1a) and phytoplankton biomass (S1 Table) to values close to zero. Overall mean (± *SD*) organic carbon pools represented by phytoplankton, bacterioplankton, POC and DOC, respectively, were 81 ± 72 (*n* = 15), 123 ± 77 (*n* = 15), 1450 ± 300 (*n* = 3) and 76 100 ± 23 000 (*n* = 16) mg C m^-2^. The ratios between the annual average phytoplanktonic and bacterial biomasses (i.e. the phytoplankton: bacterioplankton biomass ratio) was 0.5 in 2009 and 1.2 in 2011 (see biomasses and pools in S1 Table).

Distinct seasonal patterns in zooplankton δ^2^H were apparent for the different taxonomic groups of crustaceans. The calanoid (mainly *Eudiaptomus* sp.) population shifted its δ^2^H signal towards that of phytoplankton during the primary production peaks (Fig. 1b). In contrast, the δ^2^H of cyclopoids (*Cyclops* spp.) and cladocerans (mainly *Ceriodaphnia* sp.) remained close to the allochthonous source value throughout summer (even in the productive year 2011), indicating a very strong niche separation between these groups and the calanoids. These differences were also reflected in the temporal patterns of calculated allochthony, which were different for each of the studied zooplankton groups (no significant correlations between allochthony in one group and another). On average, the allochthony (mean ± *SD* of seasonal variation) was 0.22 ± 0.26 in calanoids, 0.56 ± 0.13 in cyclopoids and 0.56 ± 0.13 in the cladoceran mixed-species samples.

**Fig 2:**
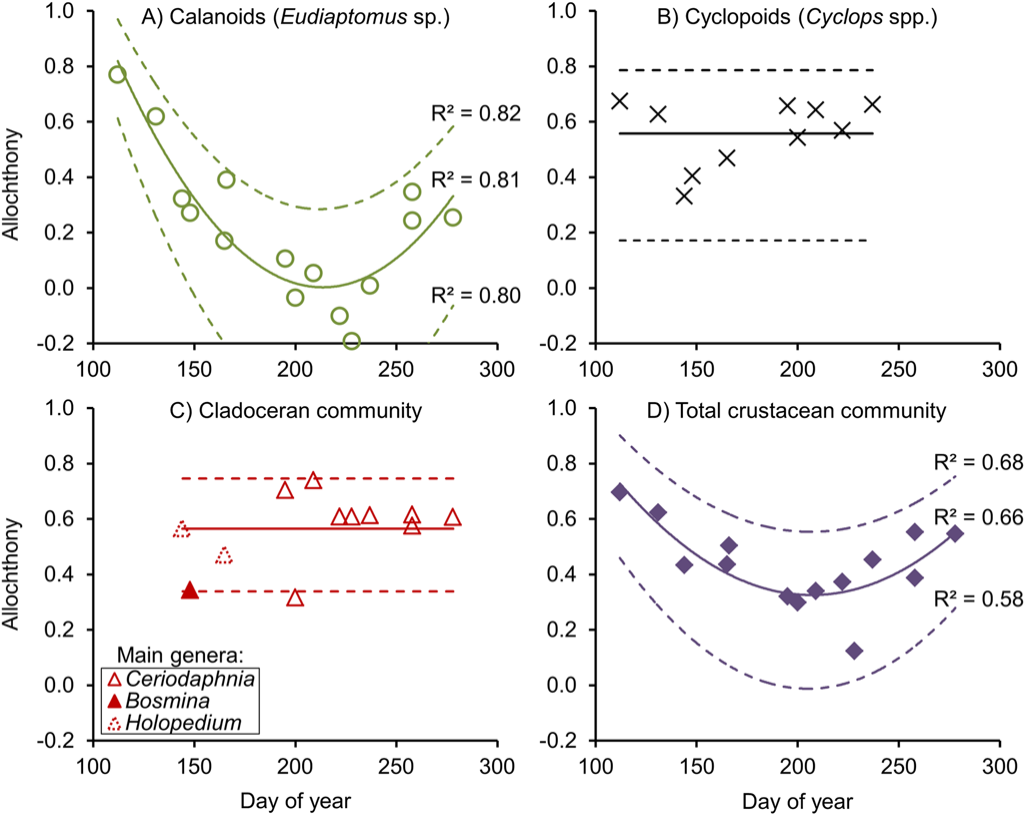
Allochthony of (A) calanoid copepods, (B) cyclopoid copepods, (C) total cladocerans, and (D) the biomass-weighted mean of the total crustacean zooplankton community, as functions of the ordinal date of lake Övre Björntjärnen 2009 and 2011. Curved solid regression lines show significant quadratic relationships (*p* < 0.01). Dashed lines show the corresponding relationships in high and low end allochthony scenarios, obtained by manipulating model parameters in all possible combinations as explained in the text. In lack of significant relationships, straight lines indicate means.

Seasonally (Fig. 2a), the allochthony in calanoids was as high as 0.6–0.8 in late winter samples (i.e. in April), but in the middle of summer (July) it was 0, before increasing again to ca 0.3 in fall. Merging all data points from 2009 and 2011, the allochthony in calanoids showed a strong (R^2^ = 0.81) quadratic relationship with day of year (DOY; Fig. 2a). The uncertainty analysis showed that this relationship had a similar strength (R^2^ = 0.80–0.82) regardless of the parameter assumptions, and that the biomass was in all scenarios mainly allochthonous (>60%) in late winter, but autochthonous (>60%) around mid-summer (Fig. 2a). In contrast, we found no significant overall seasonal patterns of allochthony (with DOY) in cyclopoids or in cladocerans (Fig. 2b-c), both showing mean allochthony of 0.56 (uncertainty range of mean 0.17–0.79 in cyclopoids and 0.34–0.75 in cladocerans, depending on model parameters). Nonetheless, the biomass-weighted mean allochthony for the whole crustacean community (Fig. 2d) showed a quadratic relationship with DOY.

**Fig 3:**
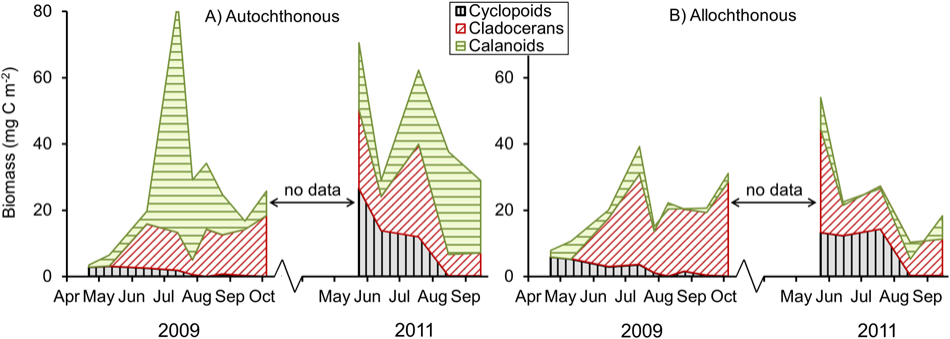
Stacked zooplankton biomasses measured at the center of lake Övre Björntjärn. The biomass for all organisms is divided into (A) an autochthonous part and (B) an allochthonous part, based on a calculation using stable hydrogen isotope data (Fig. 1b) together with an algebraic mixing model as described in the text.

The average zooplankton biomasses were 10 ± 14 mg m^-2^ for cyclopoids, 21 ± 19 mg m^-2^ for calanoids, and 28 ± 17 mg m^-2^ for cladocerans (means ± *SD*). The cyclopoid biomass showed its largest values in April to May, while calanoids and cladocerans were most abundant in summer and fall (Fig. 3). By multiplying biomass with allochthony proportions, it became evident that calanoids contributed the most (nearly 2/3) to the total pool of autochthonous biomass in the zooplankton community (27 ± 23 mg m^-2^; Fig. 3a), while allochthonous biomass (20 ± 10 mg m^-2^; Fig. 3b) was to *ca* 90% represented by cladocerans and cyclopoids. The total autochthonous fraction showed a large variability, from 3 mg m^-2^ in late winter/early spring to 83 mg m^-2^ at peak PP in summer (Fig. 3a), especially in 2009 when the calanoid population developed strongly over the season, in parallel with a shift in the calanoid δ^2^H signal towards that of phytoplankton (Fig. 1b). However, the allochthonous biomass was relatively stable over time in 2009 (Fig. 3b). Of the different zooplankton groups, only the cyclopoids showed a significant correlation between biomass and allochthony. This correlation was strongly negative (*r* = -0.89, *n* = 9, *p* < 0.01; not shown) and based on the fact that the cyclopoid allochthony increased from early to late summer (Fig. 2b), while their biomass decreased during the same periods (Fig. 3).

**Fig 4:**
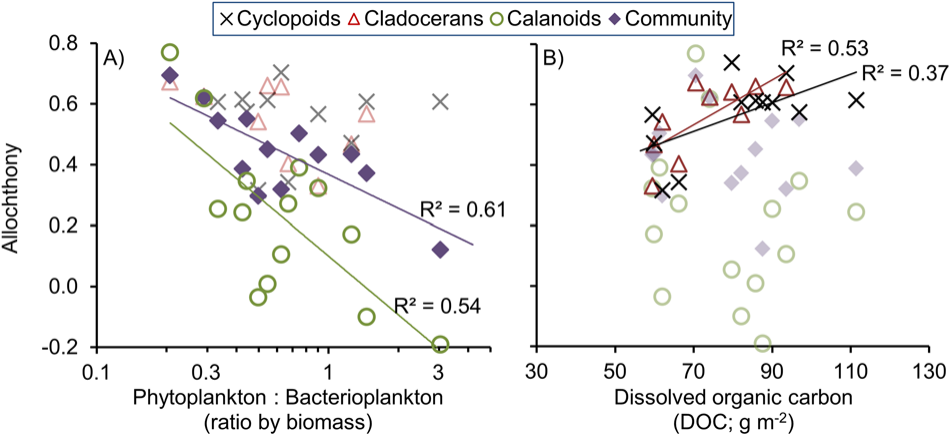
Allochthony in the different zooplankton groups as functions of (A) the ratio between phytoplanktonic and bacterioplanktonic biomass^a^ and (B) amount dissolved organic carbon in lake Övre Björntjärn. Regression lines are drawn separately for each zooplankton group which represents significant (p < 0.05) correlations. Zooplankton groups without significant correlations are shown in faded (50% less ink) symbols. Note the logarithmic scale on the x axis in banner A. ^**a**^Excluding one extreme low-end outlier on July 28, 2009, when the algal bloom was interrupted by a rain storm which suddenly flushed most of the phytoplankton biomass out of the lake (see Fig. 1a and S1 Table).

These contrasting temporal patterns could partly be explained by contrasting allochthony regulation for the different zooplankton groups. For calanoids (R^2^ = 0.54, *p* < 0.01, *n* = 14) and for the total crustacean community (R^2^ = 0.61, *p* < 0.01, *n* = 13), allochthony showed negative relationships to phytoplankton: bacterioplankton biomass ratio (Fig. 4a), when excluding an extreme outlier during the summer storm in 2009 (see explanation in Fig. 4 footnote). Thus, allochthony was linked to situations when there was lack of phytoplankton particles in relation to bacterial particles in the water column. However, without removal of the outlier, only marginally significant correlations were shown between the biomass ratio (log-transformed) and allochthony in any of the groups (Table 1). It should be noted here that the storm sampling was performed two days after the extreme rain event, which means that the zooplankton only had two days to respond to the extremely large and quick changes in resource biomasses that happened. Thus, removal of this outlier is well justified.

**Table 1:** Pearson r correlations^a^ between organic matter pools, or the phytoplankton: bacterioplankton biomass ratio, and the allochthony in different zooplankton groups in the lake Övre Björntjärn, sampled during different parts of the year.

Taxon	Bacterioplankton(mg C m^-2^)	Phytoplankton(mg C m^-2^)	Dissolved organic carbon(mg C m^-2^)	Log (Phytoplankton: Bacterioplankton)
Cladocera (*n* = 12)	-0.67* (-0.66* to -0.68*)	-0.50^(^*^)^ (n.s. to -0.51^(^*^)^)	0.61* (0.58* to 0.63*)	n.s. (n.s.)
Calanoida (*n* = 15)	n.s. (n.s.)	n.s. (n.s.)	n.s. (n.s.)	-0.48^(^*^)^ (-0.47^(^*^)^ to -0.48^(^*^)^)
Cyclopoida (*n* = 10)	n.s. (n.s.)	n.s. (n.s.)	0.73* (0.69* to 0.73*)	-0.55^(^*^)^ (n.s. to -0.61^(^*^)^)
Community (n = 14)	n.s. (n.s.)	n.s. (n.s.)	n.s. (n.s.)	-0.51^(^*^)^ (-0.50^(^*^)^ to -0.51^(^*^)^)

Values in brackets show the possible range of correlation coefficients obtained for different allochthony scenarios. The biomass ratio is log-transformed because of strong (>2) skewness.

^a^Significance: not significant (denoted n.s.);

p < 0.10 (denoted ^(^*^)^);

p < 0.05 (denoted *)

The allochthony in cyclopoids and cladocerans was not significantly (*p* < 0.05) related to the phytoplankton: bacterioplankton biomass ratios, but instead showed positive correlations to the amount of DOC in the lake (cyclopoids, R^2^ = 0.53, *n* = 10, *p* < 0.05; cladocerans, R^2^ = 0.37, *n* = 12, *p* < 0.05; Fig. 4b). In addition, allochthony in cladocerans showed a significant negative correlation to the bacterioplankton biomass and a marginally significant negative correlation to the phytoplankton biomass (see details in Table 1). The uncertainty analysis showed that these correlations had similar strengths regardless of model parameter assumptions (Table 1). On the 0.05 significance level, we found no relationships between allochthony and absolute or relative rates of PP or BP in the lake.

## Discussion

The question of whether or not freshwater organisms are associated with significant degrees of allochthony has been addressed in more than 30 different isotope mixing model studies (see compilation in [4]), yet there is poor knowledge on the controls on, and the ecological consequences of, allochthony variations across space and time. This study shows that there can be a strong niche separation between calanoid micro-crustaceans and other crustacean zooplankton during phytoplankton primary production peaks in unproductive humic lakes.

The calanoid zooplankter *Eudiaptomus* sp. made efficient use of the autochthonous phytoplankton peak production in the study lake, thereby increasing its biomass to maximum values in the middle of the summer. In support of our prediction, there was a strong negative correlation between calanoid allochthony and the abundance of phytoplankton biomass relative to bacterioplankton biomass. Hence, the calanoid population made a remarkable shift from ca 80% allochthony in April to 0% allochthony in July. This pattern agrees well with previous results from the closely (ca 15 km downstream) located humic lake Örträsket, where Meili et al. [46] found that the δ^13^C in calanoids (mainly *Eudiaptomus* sp.) was similar to allochthonous resources in March (-27‰) but switched towards more negative values overlapping with assumed phytoplanktonic δ^13^C in summer (-35‰). This switch happened even if the δ^13^C of the bulk particulate organic matter remained close to the allochthonous source (-27‰ to -30‰) throughout the seasons, suggesting that calanoids (and especially *Eudiaptomus*) is a highly specific phytoplankton grazer in the region [46]. Our study also show that the calanoids shifted back towards an allochthonous diet in fall, similar to what Rautio et al. [22] reported for the clear-water subarctic Lake Saanajärvi, northern Finland, and coherent with observed shifts among calanoids from herbivory in summer to omnivory during other seasons [47, 48].

In contrast to the study by Rautio et al. [22] cladocerans in our lake remained at high levels in allochthony irrespective of season and never shifted towards a phytoplankton diet in summer. In their study in Lake Saanajärvi, the seasonal change in levels of allocthony was connected to a shift in particle composition, with phytoplankton constituting more than a quarter of the POC pool during the PP peak [22]. In our study lake, allochthony in cladocerans probably remained high because autochthonous resources were to a much higher extent diluted by allochthonous ones in comparison to the lake studied by Rautio et al. [22]. The mean summer phytoplankton biomass in our study was only 5% of the mean POC concentration in the lake, and bacterioplankton often dominated over phytoplankton, both in terms of biomass and productivity (see S1 Table). Nonetheless, although the mean allochthony in cladocerans was relatively high (0.56), it is worth pointing out that cladocerans still showed a certain degree of selective assimilation of autochthonous organic matter, since they were not as overwhelmingly dominated by terrestrial resources as the POC pool.

In support of our hypothesis, also the cyclopoid community remained at high allochthony levels during most parts of summer, even when the phytoplankton primary production was relatively high. Further, allochthony in both cyclopoids and cladocerans, but most strongly in cyclopoids, correlated positively to DOC amounts in the lake. This, again, supports our predictions and underscores the likely importance of DOC-based food chains for cyclopoids. However, unlike the case in a recent study in Canada [4], we did not find support for a significant correlation between BP and cyclopoid allochthony, possibly because our point measurements of bacterial production were affected by considerable variability from day to day, while crustacean biomass integrates resources that have been assimilated into the food web during the recent last few weeks.

During late winter, spring and fall, all groups (including calanoids) were sharing the allochthonous resources. However, it needs pointing out that the calanoids never represented a large part of the allochthonous zooplankton biomass at any part of the year (Fig. 3b). Thus, it is not likely that the calanoids in our study lake at any time during the year represent a significant link between allochthonous resources and higher trophic levels such as fish. Furthermore, the vast overall dominance of autochthonous biomass in calanoids (Fig. 3) suggests that growth and/or reproduction were strongly limited by phytoplankton abundance and/or production. Nonetheless, previous studies have shown that ingestion of microzooplankton and detritus (both of which are associated to bacterioplankton) can be an overwintering strategy of calanoid copepods in cold climate [47, 48]. In support of these previous results, our study suggests that such an overwintering strategy can lead to extremely high levels of allochthony by the end of the winter season (April).

It may seem counter-intuitive that cyclopoids, who are selective feeders [25], continue to use allochthonous resources even under the presence of a potent food source such as that provided by phytoplankton during the summer PP peak. However, cyclopoids often act as raptorial feeders on other zooplankton [24, 25], which in our study agrees with the picture painted by the δ^15^N of the cyclopoids, indicating that the food source that these animals were using was one trophic level above the basal level. This also means that the cyclopoids not necessarily derived their terrestrial-like stable hydrogen isotope signal by directly ingesting bacteria or allochthonous POC [9], but rather by consuming organisms that, in turn, were utilizing the allochthonous basal resources. Although speculative, it is possible that such protozoan microzooplankton provided an allochthonous-based cyclopoid food resource which was as significant as that provided by obligate photoautotrophic phytoplankton. This seems especially likely in our study lake Övre Björntjärn, given the generally high bacterial production and biomass compared to phytoplankton production and biomass (Fig. 1, S1 Table), and the substantial transfer of carbon from bacteria to bacterivorous protozoa in humic lakes [49].

Considering the large seasonal variability in food sources for micro-crustaceans, especially for calanoids as shown here and previously in clear-water lakes [20], faulty assumptions regarding the fixed trophic positions can easily bias the stable H mixing model results. We here assumed a fixed trophic position, but our uncertainty analysis included a wide range of trophic position values, between 1 and 2 steps above phytoplankton (primary and secondary consumer level, respectively). Therefore, the seasonality of allochthony is well constrained within the uncertainty boundaries shown in Fig. 2.

In summary, our study shows strong contrasting allochthony patterns in calanoid and cyclopoid copepods. For calanoids, the use of allochthonous resources appeared important for sustaining a small population of active animals in the water column during late winter, but in summer the calanoids shifted to 100% herbivory, increasing their biomass several-fold by making efficient use of the pelagic primary production. The cyclopoid allochthony was high in late winter, and they most likely fed on other consumers, probably microzooplankton. Cladocerans had high levels of allochthony irrespective of season. Thus cyclopoids, together with cladocerans, can represent a significant link between DOC-based food chains and higher trophic levels in humic lakes.

## Supporting Information

S1 Table Water column integrated organic matter pools in lake Övre Björntjärn, expressed in mg C m^-2^. Areal estimates of carbon pools in bacterioplankton, phytoplankton, particulate organic carbon and dissolved organic carbon, per sampling date.
(DOCX)
