# Supplementary material for: Intraspecific Autochthonous and Allochthonous Resource Use by Zooplankton in a Humic Lake during the Transitions between Winter, Summer and Fall
Source: PLoS One. 2015 Mar 12;10(3):e0120575. doi: 10.1371/journal.pone.0120575 (PMC4357398; doi:10.1371/journal.pone.0120575)
Supplement: S1 Table — Areal estimates of carbon pools in bacterioplankton, phytoplankton, particulate organic carbon and dissolved organic carbon, per sampling date. (DOCX) [file pone.0120575.s001.docx]

**S1 Table. Water column integrated organic matter pools in lake Övre Björntjärn, expressed in mg C m^-2^.** Areal estimates of carbon pools in bacterioplankton, phytoplankton, particulate organic carbon and dissolved organic carbon, per sampling date.

| Date | Bacterioplankton | Phytoplankton | POC | DOC |
| --- | --- | --- | --- | --- |
| 4/22/2009 | 93 | 19 | - | 70 500 |
| 5/11/2009 | 244 | 71 | - | 74 100 |
| 5/28/2009 | 101 | 68 | - | 66 200 |
| 6/15/2009 | 81 | 61 | - | 61 300 |
| 7/14/2009 | 66 | 41 | - | 93 600 |
| 7/28/2009 | 110 | 10 | - | 79 700 |
| 8/10/2009 | 68 | 100 | - | 82 100 |
| 8/25/2009 | 82 | 45 | - | 85 800 |
| 9/15/2009 | 111 | 49 | - | 96 900 |
| 10/5/2009 | 105 | 35 | - | 90 000 |
| 5/24/2011 | 85 | 77 | 1150 | 59 500 |
| 6/20/2011 | 234 | 294 | - | 59 900 |
| 7/19/2011 | 315 | 157 | - | 62 000 |
| 8/16/2011 | 50 | 152 | 1450 | 87 600 |
| 9/13/2011 | 101 | 43 | 1750 | 111 400 |

Abbreviations: DOC, dissolved organic carbon; POC, particulate organic carbon
